# Paired arrangement of kinetochores together with microtubule pivoting and dynamics drive kinetochore capture in meiosis I

**DOI:** 10.1038/srep25736

**Published:** 2016-05-11

**Authors:** Gheorghe Cojoc, Ana-Maria Florescu, Alexander Krull, Anna H. Klemm, Nenad Pavin, Frank Jülicher, Iva M. Tolić

**Affiliations:** 1 Max Planck Institute of Molecular Cell Biology and Genetics, Pfotenhauerstr. 108, 01307 Dresden, Germany; 2 Max Planck Institute for the Physics of Complex Systems, Nöthnitzerstr. 38, 01187 Dresden, Germany; 3 Interdisciplinary Research Institute, Université des Sciences et des Technologies de Lille (USTL), CNRS USR 3078, 50, Avenue Halley, 59568 Villeneuve d’Ascq, France; 4 Department of Physics, Faculty of Science, University of Zagreb, Bijenička cesta 32, 10000 Zagreb, Croatia; 5 Division of Molecular Biology, Ruđer Bošković Institute, Bijenička cesta 54, 10000 Zagreb, Croatia

## Abstract

Kinetochores are protein complexes on the chromosomes, whose function as linkers between spindle microtubules and chromosomes is crucial for proper cell division. The mechanisms that facilitate kinetochore capture by microtubules are still unclear. In the present study, we combine experiments and theory to explore the mechanisms of kinetochore capture at the onset of meiosis I in fission yeast. We show that kinetochores on homologous chromosomes move together, microtubules are dynamic and pivot around the spindle pole, and the average capture time is 3–4 minutes. Our theory describes paired kinetochores on homologous chromosomes as a single object, as well as angular movement of microtubules and their dynamics. For the experimentally measured parameters, the model reproduces the measured capture kinetics and shows that the paired configuration of kinetochores accelerates capture, whereas microtubule pivoting and dynamics have a smaller contribution. Kinetochore pairing may be a general feature that increases capture efficiency in meiotic cells.

Cell division needs equal distribution of the genetic material, from the mother cell to the two daughter cells. A failure of equal distribution of the genetic material results in aneuploidy, which is the cause of severe genetic syndromes (e.g., Down syndrome) and a hallmark of cancer. In order to distribute the genetic material equally, the cell forms a spindle, a precise micro-machine made of microtubules (MTs) to which all chromosomes have to be correctly targeted. MTs interact with a chromosome via the kinetochore (KC), a large protein complex located at the centromere. MTs have to get in close proximity to the KCs in order to be able to interact with them, but the mechanisms that facilitate this process are still under debate^1^.

A model system where the targeting of the chromosomes to the spindle can be studied is meiosis I in the fission yeast *Schizosaccharomyces pombe* (*S. pombe*). At the onset of meiosis I, the two spindle pole bodies (SPBs) start to nucleate MTs, which form the spindle. At the same time, KCs are found away from the SPBs^2^,^3^. In order to get in close proximity to the KCs, MTs have to explore the space.

Several models have been proposed to explain how MTs explore the space while searching for KCs. The pioneering idea termed “search-and-capture”, proposed by Mitchinson and Kirschner in^4^, suggests that MTs nucleate at the centrosomes with a random orientation, grow and shrink to probe the space, and eventually encounter KCs. However, random probing of space by MTs while searching for multiple small targets is likely to be slow. Thus, a number of models have been introduced to describe how the capturing process can be accelerated: by a biased MTs growth toward the KC^5^,^6^, by MTs nucleated from the KC and by KC movement^7^,^8^. We have recently shown that MTs explore the space by pivoting around the SPB, which accelerates KC capture in mitosis in fission yeast^9^. All these mechanisms of KC capture contribute most likely to a different extent in different spindles.

In the present study, we combine experiments and theory to explore the relative contribution of the mechanisms that lead to KC capture at the onset of meiosis I in fission yeast. Based on our experimental results, our theoretical model includes MT angular movement around the SPB and MT dynamics. In addition, we model KCs on homologous chromosomes as a single object because we have observed that they move in a correlated manner. Our simulations show that both MT dynamics and their angular movement around the SPB accelerate KC capture. Moreover, the paired configuration of KCs on homologous chromosomes significantly increases capture efficiency.

## Results

### Movement of KCs is spatially correlated, and their capture and retrieval show specific time dynamics

At the onset of meiosis I in the fission yeast *S. pombe*, the KCs are far apart from SPBs and telomeres, and SPBs are clustered together^2^. The SPBs nucleate MTs that form the spindle. At the same time other MTs grow, probe the space, bind to KCs and pull them toward SPBs.

**Figure 1:**
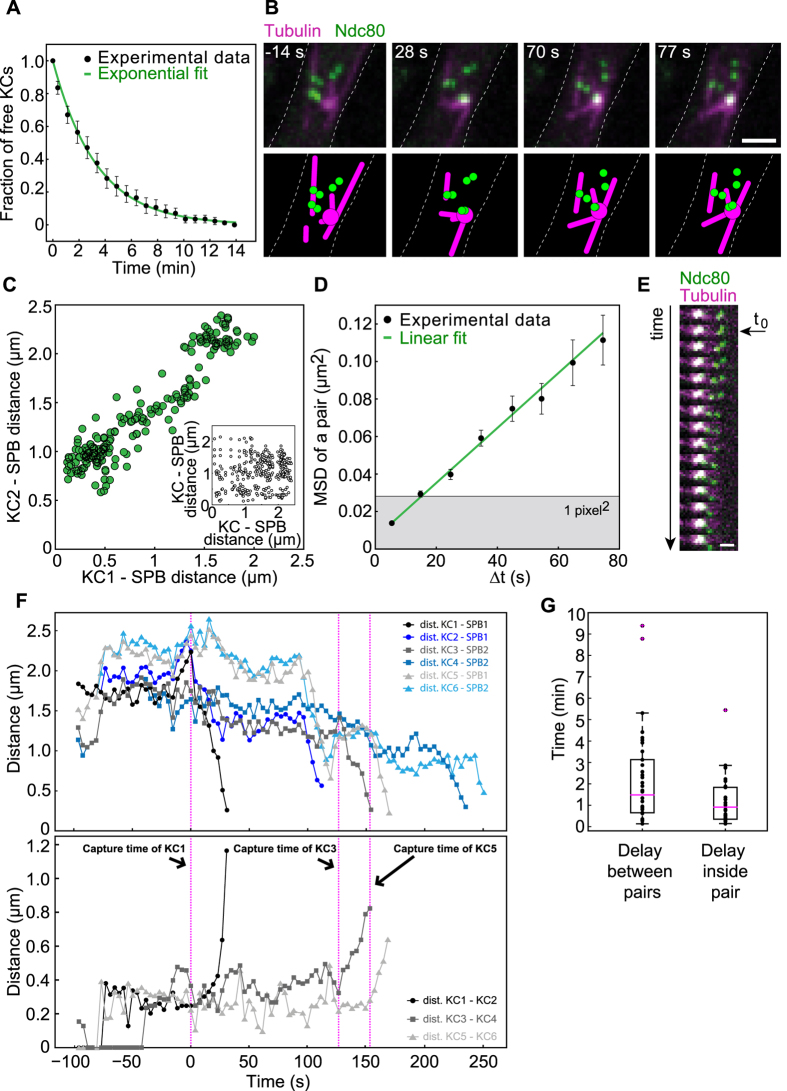
KCs move and are captured in pairs. All data shown in this figure were obtained by using the strains GC03 and GC04. The zygotes expressed α-tubulin-mCherry, shown in magenta, and Ndc80-GFP, shown in green. **(A)** Normalized averaged fraction of free KCs as a function of time (mean ± s.e.m., n = 102, time bin = 45 seconds). An exponential fit to the equation 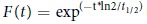 (green line) yielded a half-life t_1/2_ = 2.31 ± 0.09 minutes. **(B)** Time-lapse images and corresponding drawings representing KCs forming 3 pairs. In the second image the first pair is captured and moved to the SPB. Scale bar is 2 μm. **(C)** Correlation between the distances KC-SPB for each pair (in green). On the x-axis is the distance between the first captured KC in a pair and the SPB, and on the y-axis the distance between the second captured KC and the corresponding SPB. In the inset, no correlation between KC-SPB distances of KC’s from different pairs (black). On the x-axis is the distance between a KC and the SPB, and on y-axis is the distance between each KC from other pairs and SPB. **(D)** Mean squared displacement of the KC pair. A weighted fit to the equation MSD = 4D_KC_Δt + offset (green line) yielded a diffusion coefficient of KC pair *D*_*KC*_ = 3.9 ± 0.2 *10^−4 ^μm/s (mean ± SD, n = 51). KCs were tracked with subpixel resolution (Methods) and the center of mass of a pair was calculated. Grey denotes the area corresponding to subpixel movement. **(E)** Time-lapse images representing the capture and retrieval of a KC pair. First, one KC is captured and pulled toward the SPB (white area). The second KCin this pair does not move for about 15 seconds (4 frames), but afterwards starts to move towards the SPB. Time difference between frames Δt = 3.85 seconds. Scale bar is 1 μm. **(F)** Top: distance between each KC and the SPB. Circles, squares and triangles mark different KC pairs, see legend. Bottom: distance between the KCs from the same pair. The magenta dashed lines represent the capture event of the first KC in each pair. **(G)** Box plots of the delay capture time between pairs (left) and delay capture time between KCs inside each pair (right).

To quantify the kinetics of KC capture, we measured the decrease in number of free KCs over time. Free KCs were defined as KCs that freely move within the nucleoplasm without being connected to any MT or to SPBs. A KC was considered captured when the signals from the KC and MT overlap and the KC starts to move toward the SPBs. The time reference was chosen as the frame when the first capture was observed. For this system, the process of capturing lasts, on average, for about 12 minutes and only for one cell, we observed that the process of capturing took more than 15 minutes. The average number of the free KCs was halved within 2.31 ± 0.09 minutes (Fig. 1A), which defines the typical capture time in this system.

By live-cell imaging of cells with KCs labeled in green (Ndc80-GFP) and MTs in magenta (α-Tub-mCherry) we observed 6 KCs that seemed to be grouped in three pairs (Fig. 1B). To address this point, we followed the position of KCs and SPBs as function of time, using a custom made tracking software^10^,^11^, and we computed the distances between KCs and SPBs. We found a linear correlation between KC-SPB distances of the same pair (Fig. 1C), but no correlation between KC-SPB distances of KC’s from different pairs (inset of Fig. 1C). This result suggests that KCs are grouped in three pairs, the movement of KCs from the same pair is spatially correlated and when one pair gets in close proximity of the SPB either KC in pair is close to the SPB.

Interestingly, two types of movements of the KCs were observed, a first one that seemed to be random and a second one that was directed toward the SPBs and coincided with the moment when a KC gets in the vicinity of a MT. We first focused on the movement of KCs before the moment of capture, and to address this point we looked at the mean squared displacement (MSD) of the center of mass of a single pair. The position of the center of mass was determined from the positions of the two KCs. By computing the MSD of a single pair, we found that the movement of single pairs before capture is diffusive, with a diffusion coefficient D = 3.9 ± 0.2* 10^−4 ^ μm/s (Fig. 1D).

To determine whether and how the capture of a KC influences the movement and capture kinetics of the other KCs, we analyzed how the distances of the KCs and SPBs are changing over time. As can be observed in kymograph Fig. 1E, KCs initially move together toward the SPBs, but after a while only one KC continues the movement and the second one remains behind and moves randomly until it is also captured. The movement of a KC that occurs after the capture is known as “KC retrieval”^12^,^13^,^14^. In Fig. 1F, we show the distances between each KC and the SPB where it was retrieved. Each pair of KCs is depicted with a different color. As can be observed for all three pairs, although the retrieval of the first KC in a pair determines the movement of the second KC from the same pair closer to the SPB, the distance between KCs from the same pair increases (Fig. 1F bottom). The density of MTs is higher closer to SPBs and this is expected to increase the chance that the second KC in a pair is captured faster. We calculated the delay of the capture time between pairs and between KCs inside of the same pair (Fig. 1G). The delay in capture between pairs was more than 2 minutes and inside each pair for about 1 minute. These findings suggest that the capture of a KC influences the movement and the capture kinetics of the KC within the pair, but not the capture kinetics of the other KCs.

### Highly dynamic MTs perform angular movement to explore the entire nucleus

**Figure 2:**
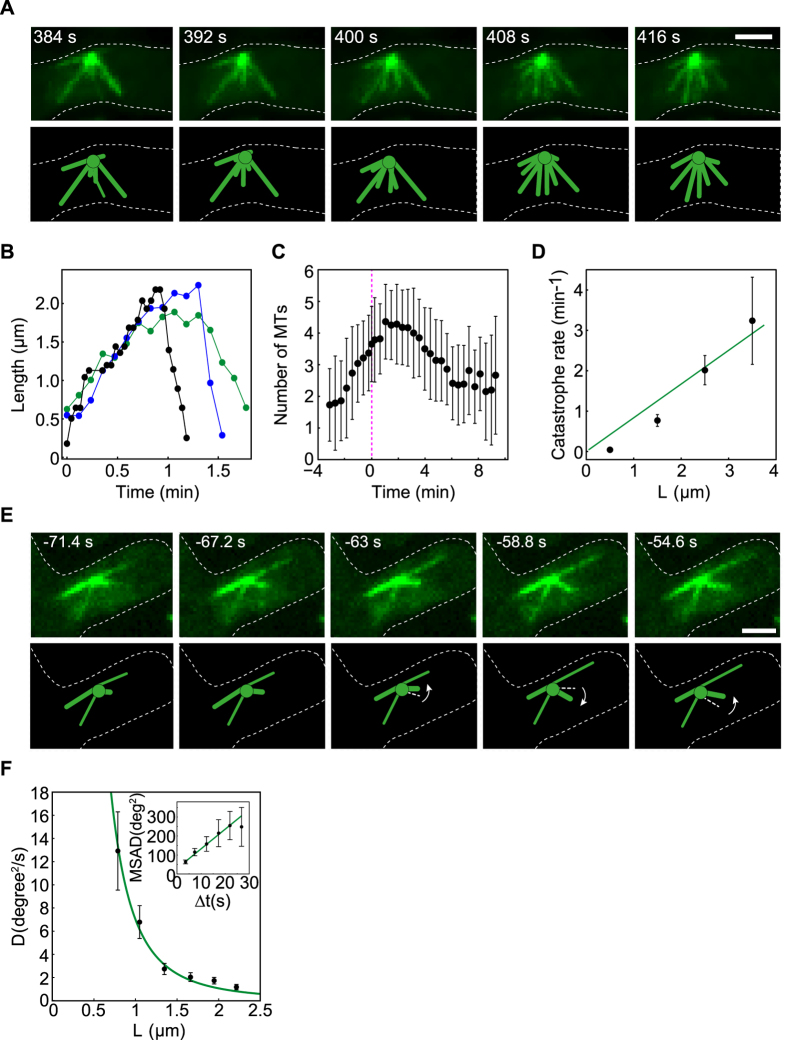
MTs perform pivoting and are highly dynamic during meiosis I. (**A**) Time-lapse images and corresponding drawing of MTs at the time interval with the highest turnover. (**B**) Example of the length variation of three MTs over time. (**C**) Number of MTs as a function of time (mean ± SD, zygotes n = 23. The SD was computed assuming that the catastrophes obey a Poisson distribution.). Time 0 represents the frame when the first capture was observed. (**D**) Catastrophe rate as a function of MT length (mean ± SD, n = 69). The green line is a linear fit (*k*_*cat*_ = α * L). (**E**) Time-lapse images and corresponding drawing of MTs that perform angular motion. **(F**) Dependence of the MTs angular diffusion coefficient on MT length (mean ± s.e.m., n = 70). Inset: mean squared angular displacement for MTs of length *L* = 1.49 ± 0.18 μm (mean ± s.e.m, n = 53).

Additionally to the kinetics of the KCs we investigated the dynamics and the movement of MTs. To address the dynamics of the MTs, we performed live-cell imaging of cells with MTs labeled in green (α-Tub-GFP) and, as can be observed in the time-lapse from Fig. 2A, in only 30 seconds MTs covered most parts of the nucleus. We found a MT’s growth rate *v*_*g*_ (2.4 ± 0.7 μm/min) consistent with^9^,^13^,^15^,^16^ and shrinkage rate, *v*_*s*_, almost three times higher (6.4 ± 1.9 μm/min). Unlike as has been reported in^9^, the life of most MTs (78%) consisted of two periods, growth and shrinkage, and only for few of them a pausing period was observed (Fig. 2B). We then computed the MT catastrophe frequency for different MT lengths. We found that MTs undergo catastrophe with a rate that increases linearly^17^ with their length with a slope of α ~1.1 (μm*min)^−1^ (Fig. 2D). MTs lived on average for ~1.2 min and the averaged maximum length was *L* = 2.22 ± 0.66 μm. They spend *L/v*_*g*_ ≈ 0.9 min in the growth phase and *L/v*_*s*_ ≈ 0.3 min in the shrink phase. These calculations show that MTs have short pausing intervals and a fast turnover during meiosis I.

To understand how the fast turnover of the MTs influences the process of capture, we measured the number of MTs over time. At the end of the meiotic prophase few intra-nuclear MTs are barely visible. Their number increases in time and reaches a peak of about 5–6 MTs/cell in the first minute after the first capturing event occurs (Fig. 2C). This high number of MTs is maintained for about 2 minutes increasing the chances that a MT finds and attaches to a KC. This timing coincides with the time when the number of KCs was halved. Although the MTs shrink rapidly a high number of MTs is maintained constant by fast turnover of the MTs.

To complete the picture of the behavior of MTs, we analyzed their movement. As previously was reported in^9^, we also observed that intra-nuclear MTs change their orientation with time in meiosis I (Fig. 2E). We calculated the mean squared angular displacement (MSAD)^18^, for MTs with an average length of 1.49 ± 0.18 μm, to distinguish whether MTs angular movement is directed or random. We found that the MSAD increases roughly linearly with time (inset of Fig. 2F) and we know that such linear relationship is associated with unbiased random movement. The corresponding angular diffusion coefficient of MTs was calculated from the slope after fitting the experimental data. Furthermore we found that the angular diffusion coefficient of the MTs decreases for longer MTs (Fig. 2F). This is in agreement with the Brownian angular motion of a thin rigid rod^19^,^20^,^21^. Taken all together, our observations regarding MT’s movement suggest that MTs are freely jointed to SPBs, pivot around them and that the movement of MTs is Brownian.

### A simple model can explain the observed captured times of the KCs

**Figure 3:**
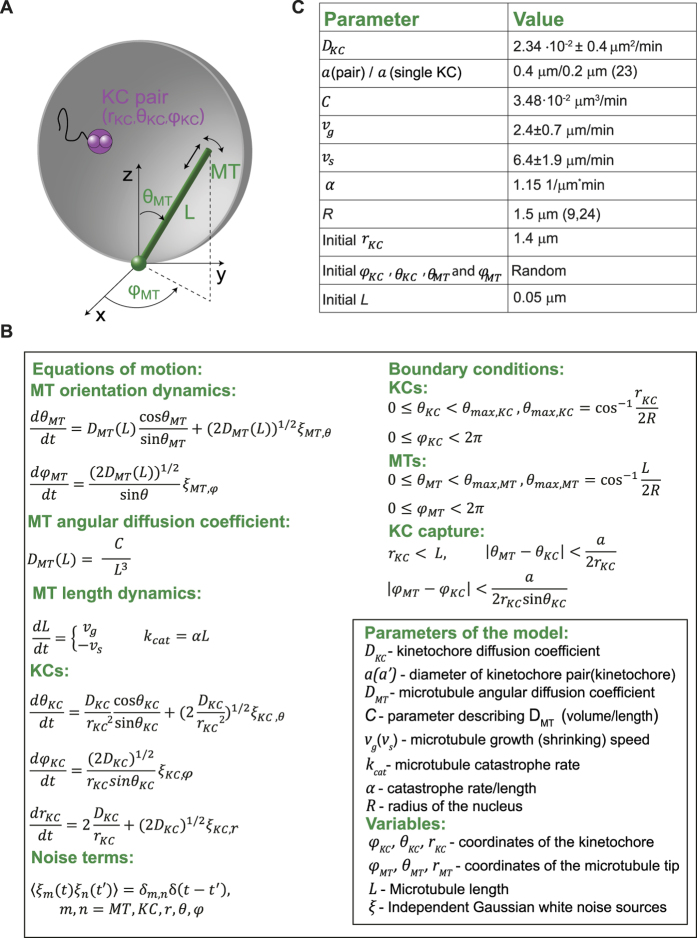
Description of the model for simulating KC capture. (**A**) Geometry of the model. A MT is described as a stiff rod, which is freely jointed to the SPB and exhibits dynamic instability. A KC pair is described as a single sphere with a radius which is twice as large as that of a single KC. **(B)** Model equations: The first column shows the equations of motion for the KCs and the MTs, the length dependence of the MT diffusion coefficient and the equations for MT growth, shrinkage and catastrophe rate. The second column shows the boundary conditions for MTs and KCs and the condition defining a KC capture event. (**C**) Table of model parameters. The values of *D*_*KC*_ , *v*_*g*_, *v*_*s*_, and the initial value of *r*_*KC*_ were measured in this work. The value for *C* was obtained by fitting the measured values of the MT angular diffusion coefficient as a function of length to the expression of the angular diffusion coefficient of a rigid rod. The parameter α was computed by fitting the measured length dependence of the MT catastrophe rate with a straight line passing through the origin. The value for *a* was obtained by doubling the value for a single KC that is given in^26^ and the value for *R* is taken from^33^.

To understand the mechanisms that lead to the observed KC capture times we developed a stochastic model that takes into account MT pivoting around the SPB (as was described in our previous work^9^, MT dynamics and paired movement of the KCs. The nucleus is described as a sphere of radius 1.5 μm corresponding to the nuclear membrane^9^. We consider that the two SPBs are located together at one pole of the sphere and acting as a single MT nucleation source. This choice is based on our observation that two SPBs are close to each other during most of the capturing process. MTs are described as thin, rigid rods that can grow, shrink and perform angular diffusion while fixed with one end at the SPB (Fig. 3A). They can switch from growth to shrinkage (*catastrophe*) with a probability per unit time that increases linearly with MT length, as shown by our experiments (Fig. 2B) and as was observed previously in^17^,^22^. The model does not include the possibility of switching from shrinkage to growth (*rescue*), since we did not observe this in our experiments. The simulations were performed using a constant number of MTs, so if one MT shrinks until its length equals zero it immediately starts growing again, with a new random orientation. A pair of KCs was described as a single rigid sphere that has a radius twice as large as that of a single KC. To check that this description can be used for studying capture we plotted the experimental fraction of free KC pairs as a function of time and observed that it obeys a kinetics that is very similar to that of the capture of single KCs.

**Figure 4:**
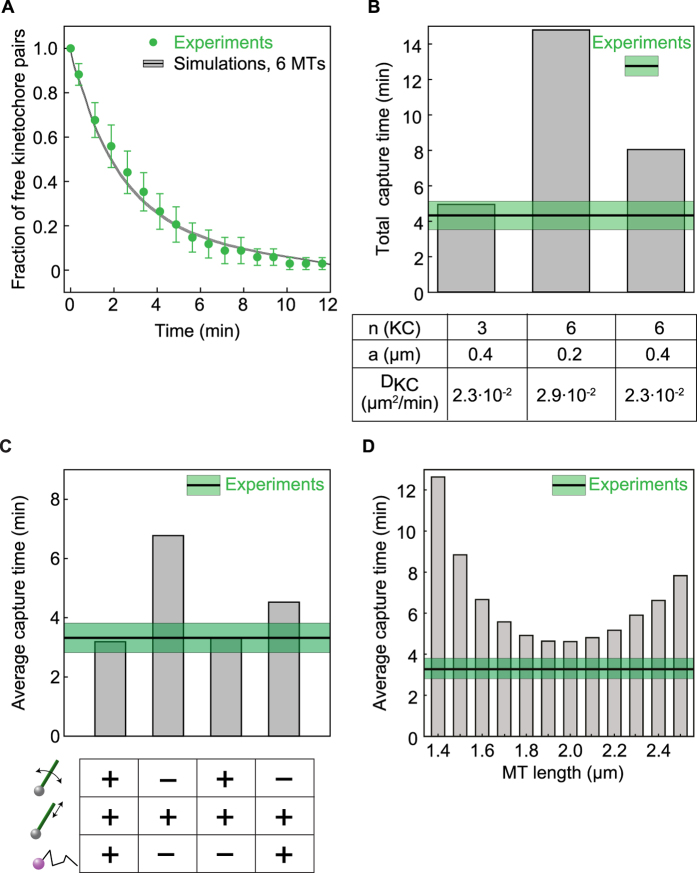
Model predictions. (**A**) Simulated and measured fraction of free KCs. The green dots are the experimental values for pairs (capture time of the first KC in a pair; mean ± s.e.m., n = 51). The black line shows simulations for the case with 6 MTs and the other parameters given in Fig. 3C and the corresponding standard error of the mean. All simulation results are averaged over 10000 runs with random initial orientations for both MTs and KC pairs. **(B)** Predictions of the model for the total KC capture time (or moment of the capture of the last KC) in the case where the three KC pairs were replaced by six free KCs. First bar: simulations with the measured parameters (Fig. 3C); second bar: simulations for six unpaired KCs; third bar: simulations for six pairs of KCs. The parameters that were changed with respect to the measured values are given under each bar. Note that *D*_KC_ = 2.9·10^−2 ^ μm^2^/min was obtained by measuring the diffusion coefficient of KCs that were unpaired, i.e., their motion was not correlated with that of any other KC. The green area marks the experimental values (mean ± s.e.m.). The capture time for a pair of KC was computed as the time needed to capture the first KC in that pair. **(C**) The effect of diffusion on the average time of KC capture (mean first passage time averaged over each KC). The simulations were performed using the measured parameters (first bar); D_MT_ = 0, D_KC_ = 0 (second bar); D_KC_ = 0 (third bar); D_MT_ = 0 (fourth bar), as shown schematically under each bar. All other parameters were the same as in Fig. 3C. All simulations are for 6 MTs. The green area marks the experimental values (mean ± s.e.m.). **(D)** Average capture time (mean first passage time averaged over each KC) as a function of MT length for the case in which dynamic instability is switched off (MT length is constant). All the other parameters are as in Fig. 3C. All simulations are for 6 MTs. The green area marks the experimental values (mean ± s.e.m.).

The motion of both KCs and MTs was described using Langevin’s equations in spherical coordinates (shown in Fig. 3B). These equations describe a realization of the stochastic Brownian motion of objects immersed in a fluid by considering the effect of the fluid to be similar to that of a random force exerted on the particle. We solve these equations by numerical integration using the Euler method. When integrating the equations, we consider that the motion of the KCs and MTs is confined within the sphere (the boundary conditions are given in Fig. 3B). When a KC touches the membrane, it is reflected back towards the center of the nucleus. When the tip of a MT grows until it touches the nuclear membrane (that is, the boundary is reached due to a change in *L*) it slides along it until it does not touch it anymore. If a MT hits the boundary by pivoting (due to a change of one of the angular variables), it is reflected towards the center of the nucleus. The exact equations used to implement the boundary conditions in the simulations are given in the supplementary text. A KC capture event was considered to occur when the MT overlaps with the volume of the KC, according to the equations in Fig. 3B. Note that this allows for lateral capture. After a KC is captured by a MT, that MT undergoes catastrophe and starts to shrink, carrying the KC to the SPB. The parameters of the model have values that we measured experimentally, with two exceptions: the radius of the kinetochore, which is taken from^23^, and the radius of the nucleus, which is taken from^24^. The parameters of the model are given in Fig. 3C. The initial configuration was set by putting the MTs’ lengths to zero and positioning the KCs close to the equator of the nucleus, as measured at the onset of meiosis I. The details of the initial conditions and the parameters of the model are given in Fig. 3C. The model was tested by computing the number of free KCs as a function of time for increasing numbers of MTs. The best agreement with the experimental data is obtained for 6 MTs (Fig. 4A, S1A), which is a MT number that is in good agreement with what we observed experimentally (Fig. 2C). One has to note that in the experimental images MTs that have a length below 0.75 μm are not always observed, while they are always included in the simulation MT count. In figure S1A we show the average KC capture time (which is equivalent to the mean first passage time for each MT-KC encounter, averaged for all KCs) for other values of the MT number. As for the experimental data, time zero is considered when the first KC pair is captured, so the average is taken over the remaining two pairs. We have then checked that the model we used for describing MT catastrophe does not influence our results (see Supplementary text and supplementary figures S1A–F and S2A–C for a detailed discussion). The results above validate our model and show its robustness.

We then simulated the capture of six unpaired KCs instead of three KC pairs and observed that the average capture time has almost doubled. The main reason for this is that a pair has a larger surface available for interaction with MTs, thus facilitating attachment. This is also supported by the prediction that when increasing the number of KCs without changing their properties the average capture time stays the same, but the total capture time (or the lifetime of the last kinetochore that is being captured) increases from 4.81 minutes to 7.76 minutes (Fig. 4B). This result predicts a second crucial contribution to capturing, which has not been suggested before: the pairing of KCs.

The theoretical model was then used to make predictions regarding the mechanisms that contribute to KC capture and to estimate their contributions. Simulation parameters were changed in turn, as follows: (i) D_MT_ = 0, D_KC_ = 0 (ii) D_KC_ = 0, and (iii) D_MT_ = 0. All the other parameters were kept as in Fig. 3C. Case (iii) corresponds to the “search and capture” model of Mitchinson and Kirchner^4^. We denote by case (0) the simulations that were done using the measured parameters. The results of all cases are shown in Fig. 4C. When both the angular diffusion coefficient of the MTs and the diffusion coefficient of the KCs were put to zero (case (i)) the predicted average capture time increased with a factor of ~2.75 with respect to case (0). This could be explained by the fact that, when neither the MTs nor the KCs are diffusing, the MTs have to reach for the fixed KCs just by growing. Time is lost when a MT is not growing directly towards a KC, so the search and capture mechanism alone is the slowest search strategy. In the simulations in which only one of the two diffusion coefficients is put to zero (cases (ii) and (iii)) the average capture times were smaller than the one of case (i) but larger than in case (0). This points to the fact that at least one component of random motion (Brownian motion of either MTs or KCs) is needed for the capture process. This can be explained as follows: a MT, which does not perform Brownian motion, only captures a fixed KC by growing directly towards it. However, if the KCs move even with a small speed their motion will contribute to the capture because it increases the chances of an encounter with the MT. Differently put, the difference in capture time between the these situations is strongly related to how much space is sampled in a given time by MTs and KCs together. The presence of Brownian motion maximizes this time. Similarly, if the KC is fixed but the MT pivots, the search is accelerated because a MT that does not grow directly towards a KC can still capture it through a change in direction. Actually, in the presence of MT pivoting the effect of the KC’s motion becomes negligible (case (ii) compared to case (iii)), because pivoting is faster than the diffusion of KCs, so the latter becomes just a small addition to the amount of space that is being covered.

We also performed simulations in which the MT length is kept constant at different values ranging from 1.4 to 2.5 μm. The results of these simulations are shown in Fig. 4D. The average capture time decreases when the MT length is increased until it reaches an optimal value at ~2 μm and then it starts increasing again. This happens because the amount of space sampled by the MT is determined by two factors: its length and its angular velocity. A long MT swipes more space to search for the KC, but it moves slower, while a short one pivots fast but samples less space. MTs shorter than 1.4 μm (the initial distance between the KC and the SPB) will take a very long time to capture KCs. This is because they cannot reach a KC by pivoting, so they have to ‘wait’ until a KC diffuses towards them. Taken together, the results from Fig. 4C,D suggest that MT dynamic instability and angular motion are important contributors to KC capture.

In order to quantify the contribution of pivoting to the capturing process we computed the fraction of lateral capture events. We considered a capturing event to be lateral capture if at the moment of capture the following condition is fulfilled:


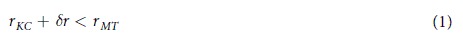


Using *δr* equals the radius of the KC, we obtained that approximately half of the kinetochores are captured laterally. Under the assumption that all lateral capture events occur due to pivoting, and all the tip capture events occur due to dynamic instability, this implies that the two mechanisms have an equal contribution. This assumption could, however, underestimate the contribution of pivoting. Using *δr* = 8 nm (the length of a tubulin) the ratio between the number of tip capture events and lateral capture events switches to 0.32. These values show that pivoting is indeed important for KC capture.

## Discussion

**Figure 5:**
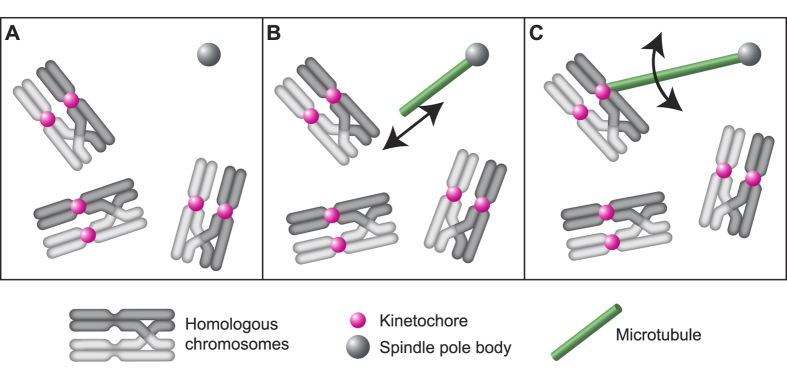
Mechanisms of KC capture. (**A**) KCs (pink) are organized in pairs on homologous chromosomes (dark and light grey). (**B**) MTs (green) grow from the SPB (grey circle) and exhibit dynamic instability. (**C**) MTs perform angular motion around the SPB. All three mechanisms together accelerate KC capture.

At the onset of mitosis and meiosis, MTs capture KCs to incorporate them into the spindle, which is required for the proper segregation of chromosomes. The mechanisms of KC capture and their relative contribution in different cell types are still not clear. We have shown that at the beginning of meiosis I in fission yeast the paired configuration of KCs on homologous chromosomes significantly increases capture efficiency (Fig. 5A). This is a novel mechanism that accelerates capture, which is specific to meiosis I. Our experiments have shown that the KCs in each of the 3 pairs, which are presumably located on a pair of homologous chromosomes, move in a correlated manner. This is most likely a consequence of the fact that homologous chromosomes are connected by a reciprocal recombination (chiasmata) at the end of meiotic prophase and the onset of meiosis I^25^. The paired configuration of KCs on homologous chromosomes may contribute to the acceleration of the capture process in two ways. First, when one KC in a pair is captured by a MT and starts to move towards the SPB, this movement brings the other KC in the same pair closer to the SPB (Fig. 1G), where the density of the MTs is higher and hence the probability of capture is also higher. Second, two KCs on a pair of homologous chromosomes provide a larger surface that has to be targeted by the MTs than a single KC, resulting in a shorter total capture time, as predicted by our model (Fig. 4B). Even though in our model a pair of KCs is described as a single large KC, the model could be extended to describe a pair of KCs as 2 KCs connected by a viscoelastic element. The prediction that the paired configuration of KCs accelerates KC capture could be tested experimentally by measuring the capture kinetics in cells lacking meiosis-specific proteins responsible for the formation of double-strand breaks and chiasmata, such as Rec12^26^. In these cells, homologous chromosomes are not organized as 3 bivalents, but as 6 univalents that are not connected with each other^27^,^28^. Thus, we expect that all 6 KCs would move independently in these cells, and our model predicts a roughly 3 times longer total capture time for this scenario.

The second mechanism that contributes to KC capture in meiosis I is MT dynamics (Fig. 5B). We observed that at the onset of meiosis I several MTs grow from the SPB, similarly as in^3^. These MTs spend most of their lifetime either growing or shrinking (Fig. 2B), thus they are highly dynamic. The dynamics of MTs was the first suggested mechanism of KC capture^4^. Indeed, our results show that the highest number of MTs is reached when the capture starts and it is maintained constant for about 3 minutes (Fig. 2C). This is similar to the time in which half of the KCs are captured. MTs in meiosis I are more numerous than in mitosis (4–5 in meiosis versus 2–3 in mitosis, on average) and more dynamic than MTs in mitosis, which spend ~70% of their lifetime at a constant length^9^. Future work will identify the molecular mechanisms that differentially regulate MT number and dynamics during meiosis and mitosis.

Finally, angular movement of MTs around the SPB accelerates KC capture (Fig. 5C). We have recently shown that this is the major mechanism underlying KC capture in mitosis in fission yeast, during spindle re-assembly after cold-induced disassembly^9^. Similarly, angular movement of astral MTs accelerates their search for cortical anchor sites during mitosis in budding yeast^29^. In general, angular movement allows MTs to explore space laterally rather than by the tip only, thereby accelerating their search for various targets such as KCs^1^. As in mitosis^9^, MT angular movement in meiosis I is random and characterized by a similar angular diffusion coefficient. Thus, MT movement in meiosis I is likely thermally driven as the movement in mitosis^9^. Our model predicts that the contribution of the angular movement of MTs to KC capture is the largest if MTs are roughly 2 μm long, which matches the experimentally measured values of MT length. It would be interesting to identify the mechanisms of MT length determination at the onset of meiosis I and to experimentally test the effect of altered MT length on the kinetics of KC capture. Ultimately, it would be important to understand what makes a particular combination of capture mechanisms suitable for the settings of meiosis or mitosis in fission yeast, as well as to what extent these mechanisms contribute to KC capture in other cell types.

## Methods

### Cells culture

*S. pombe* strains used in this work are listed in Table 2. Cells were grown on suitable agar plates at 25 °C. Zygotes were obtained from cells of opposite mating types (h+ and h−). Half-toothpick of h+ and half- toothpick of h−were suspended in 100 ml of Edinburgh Minimal Medium minus Nitrogen (EMM-N) and spotted on to Malt Extract Agar (MEA) plates. The plates were then incubated overnight, 12–16 hours, at 25 ± 0.5 °C. For imaging, a loop full of cells from the MEA plate was resuspended in 100 ml of EMM-N. The resuspended cells were transferred to a lectin-coated (L2380, Sigma-Aldrich, St Louis, MO, USA), 35 mm (No1.5) glass bottom culture dish (MatTek Corporation, Ashland, MA, USA) and allowed to stick to the glass for 5–10 minutes. Free cells were removed by washing with EMM-N and the petri dish was filled with 2 ml of EMM-N.

### Generation of *S. pombe* strains

GFP provides a higher signal-to-noise ratio than the available red fluorescence proteins (e.g. tdTomato and mCherry) and a lower bleaching rate during live cell imaging, allowing for more accurate tracking of tagged structures through a larger number of time-lapse images. Thus, we used strains expressing Ndc80-GFP to measure the kinetics of the KCs and strains expressing GFP-alpha2-tubulin (GFP-atb2) to measure the dynamics and kinetics of the MTs. We cannot exclude small differences in MT dynamics between the strains we used due to different tagging. However, here we measured MT dynamics in a strain expressing GFP-atb2, and these data are in agreement with the previously reported^16^ MT dynamics in strains expressing mCherry-atb2. Strain KI011 was obtained by crossing SV54 with strain KI006. C-terminus tagging of Ndc80 with GFP was performed by using the polymerase chain reaction (PCR) gene-targeting method^30^. The open reading frame of GFP and a KanMX resistance-cassette for selection were integrated at the C-terminus of the original gene locus of Ndc80 by homologous recombination. The primers were designed using the web tool http://www.bahlerlab.info/resources/31. The tagging-plasmid pFA6a-GFP-KanMX was used as a template (gift from K. Sawin, University of Edinburgh, UK). The product of the PCR reaction was purified using the QIAquick PCR Purification Kit (QIAGEN, Hilden, Germany), then concentrated by isopropanol precipitation with the pellet being resolved in 10 mM Tris-Cl, pH 8.5. To create GCAK1, the strain MJ95 (mCherry-atb2-hphMX leu1 ura4-D18, h-, gift of M.Sato) was transformed with 15 μg of the purified PCR product by electroporation at 1.5 kV using an Eppendorf Electroporator 2510 (Eppendorf)^32^. Ndc80-GFP-KanMX expressing strains were selected by growth on YE + supplements + hygromycin + kanamycin medium. GC03 and GC04 were obtained by crossing SI661 (Sid4-mCherry-NatMX, gift of Silke Hauf, Virginia Tech, USA) with GCAK1.

### Time-lapse live cell imaging

Cells were also imaged and manipulated using an Olympus IX71 inverted microscope with a Yokogawa CSU10 spinning-disc scan head (Yokogawa Electric Corporation) equipped with a fast piezo objective z-positioner (PIFOC, Physik Instrumente GmbH & K.G.) and an Olympus UPlanSApo × 100/1.4 NA oil objective (Olympus). For cells expressing GFP and tdTomato/mCherry, we performed sequential imaging and images were acquired at a 2.1–4.7 s time intervals. For excitation, a sapphire 488 nm solid-state laser (75 mW; Coherent) and a Jive 561 nm solid-state laser (75 mW; Cobolt) were used for GFP and tdTomato/mCherry, respectively. The laser intensity was controlled using the acousto-optic tunable filter inside the Andor Revolution Laser Combiner (ALC, Andor Technology). The emission wavelength was selected using respective emission filters BL 525/30 (Semrock) and ET 605/70 (Chroma) mounted in a fast, motorized filter wheel (Lambda 10B, Sutter Instrument Company). The images have a *xy*-pixel size of 168 nm and the z-distance between optical sections was 500 nm. The system was controlled by Andor iQ software version 1.9.1 (Andor Technology).

### Image processing and data analysis

Measurements of MT and KC positions were performed in the maximum-intensity projections of the z-stacks. Measurement of positions along the z axis were not performed because the corresponding point-spread function of the microscope is about 800 nm, which is roughly half of the length of a typical MT. An estimate of the systematic error resulting from two-dimensional measurements was presented in^9^. Maximum-intensity projections were calculated with ImageJ (National Institutes of Health) using the plug-in Stacks-Z-function—Grouped ZProjector. The colour-merge images were obtained by overlay of projections in green and magenta channels using the plug-in Colour functions—Colour merge. MT tips were tracked manually in the maximum-intensity projections using the plug-in Particle analysis—Manual tracking. Custom software^10^ was used to determine the SPBs and KCs position in the maximum-intensity projections. Data analysis and plots were performed using scripts written in MATLAB (The Mathworks).

## Additional Information

**How to cite this article**: Cojoc, G. *et al*. Paired arrangement of kinetochores together with microtubule pivoting and dynamics drive kinetochore capture in meiosis I. *Sci. Rep.* **6**, 25736; doi: 10.1038/srep25736 (2016).

## Supplementary Material

Supplementary Information

Supplementary Video 1

Supplementary Video 2

Supplementary Video 3
