# Corrigendum: Paired arrangement of kinetochores together with microtubule pivoting and dynamics drive kinetochore capture in meiosis I

**DOI:** 10.1038/srep33774

**Published:** 2016-09-22

**Authors:** Gheorghe Cojoc, Ana-Maria Florescu, Alexander Krull, Anna H. Klemm, Nenad Pavin, Frank Jülicher, Iva M. Tolić

Scientific Reports 6: Article number: 2573610.1038/srep25736; published online: 05 11 2016; updated: 09 22 2016

In this Article, the Acknowledgments section is incomplete.

“We thank M. Sato, S. Hauf, G. Rödel and the Yeast Genetic Resource Center for strains and plasmids; B. Schroth-Diez, Bert Nitzsche and Jan Peychl from the Light Microscopy Facility of MPI-CBG; V. Ananthanarayanan, M. Coelho, P. Delivani, D. Ramunno-Johnson and N. Maghelli for discussions and advice; I. Šarić for the drawings; Human Frontier Science Program (HFSP) for financial support. A.-M.F. thanks the MPG for support by a postdoctoral grant through the MPG-CNRS GDRE “Systems Biology.”

should read:

“We thank I. Kalinina, M. Sato, S. Hauf, G. Rödel and the Yeast Genetic Resource Center for strains and plasmids; B. Schroth-Diez, Bert Nitzsche and Jan Peychl from the Light Microscopy Facility of MPI-CBG; I. Kalinina, V. Ananthanarayanan, M. Coelho, P. Delivani, D. Ramunno-Johnson and N. Maghelli for discussions and advice; I. Šarić for the drawings; Human Frontier Science Program (HFSP) for financial support. A.-M.F. thanks the MPG for support by a postdoctoral grant through the MPG-CNRS GDRE “Systems Biology.”

