# Supplementary material for: Paired arrangement of kinetochores together with microtubule pivoting and dynamics drive kinetochore capture in meiosis I
Source: Sci Rep. 2016 May 11;6:25736. doi: 10.1038/srep25736 (PMC4863148; doi:10.1038/srep25736)
Supplement: Supplementary Information [file srep25736-s1.pdf]

**Supplementary Information**

# Paired arrangement of kinetochores together with microtubule pivoting and dynamics drive kinetochore capture in meiosis I

Gheorghe Cojoc, Ana-Maria Florescu, Alexander Krull, Anna H. Klemm, Nenad Pavin, Frank Jülicher, Iva M. Tolić

## 1. Equation of motion integration algorithm

The equations of motion given in figure 3B of the main text are stochastic, with the exception of the one that gives the variation in time of the MT length $L_{MT}$. $L_{MT}$ evolves according to a deterministic equation which is integrated numerically as follows:

$$L_{MT}^{(n)} = L_{MT}^{(n-1)} + v_g \Delta t \quad \text{(S1)}$$

$$L_{MT}^{(n)} = L_{MT}^{(n-1)} - v_s \Delta t \quad \text{(S2)}$$

for growing and shrinking MTs, respectively.

The stochastic equations of motion for both MTs and KCs in the form given in figure 3B correspond to the Ito interpretation of the stochastic integral (1) (for a detailed discussion, see reference (2). However, here the equations for MT orientation have an important difference compared to the case discussed in reference (1): because of the occurrence of catastrophes, there is a variation in the expression of the angular diffusion coefficient with stochastic changes due to switching between growing and shrinking states. This switch is independent of the angle variables $\theta_{MT}$ and $\varphi_{MT}$, but depends on MT length. In this case, the problem corresponds to integrating a set of Langevin equations on a series of intervals $[0,t_1),[t_1,t_2), \ldots , [t_k,t_{k+1})$ using the standard Euler-Maruyama integration algorithm (3). The $t_k$s denote the moment of occurrence of catastrophes, which are stochastic but can be defined a priori. We generate these times by drawing a random

number and comparing it with the probability to have a catastrophe in the next time step. If the random number is smaller than this probability, the catastrophe occurs.

When integrating the Langevin equations we express a variable at integration step *n* as a function of its value at step (*n-1*) as:

$$\theta_{MT}^{(n)} = \theta_{MT}^{(n-1)} + D_{MT}^{(n-1)}(L^{(n-1)})\Delta t \frac{\cos\theta_{MT}^{(n-1)}}{\sin\theta_{MT}^{(n-1)}} + \sqrt{2D_{MT}^{(n-1)}(L^{(n-1)})\Delta t} W_{\theta,MT} \quad \text{(S3)}$$

$$\varphi_{MT}^{(n)} = \varphi_{MT}^{(n-1)} + \frac{\sqrt{2D_{MT}^{(n-1)}(L^{(n-1)})\Delta t}}{\sin\theta_{MT}^{(n-1)}} W_{\varphi,MT} \quad \text{(S4)}$$

where $\Delta t$ is the integration time step and $W_{\theta,\ MT}$ and $W_{\varphi,\ MT}$ are normally distributed random numbers, drawn independently and uncorrelated for each equation. Similarly, for the KCs we have:

$$\theta_{KC}^{(n)} = \theta_{KC}^{(n-1)} + \frac{D_{KC}}{\left(r_{KC}^{(n-1)}\right)^2}\Delta t \frac{\cos\theta_{KC}^{(n-1)}}{\sin\theta_{KC}^{(n-1)}} + \sqrt{2\frac{D_{KC}\Delta t}{\left(r_{KC}^{(n-1)}\right)^2}} W_{\theta,KC} \quad \text{(S5)}$$

$$\varphi_{KC}^{(n)} = \varphi_{KC}^{(n-1)} + \frac{\sqrt{2\frac{D_{KC}\Delta t}{\left(r_{KC}^{(n-1)}\right)^2}}}{\sin\theta_{KC}^{(n-1)}} W_{\varphi,KC} \quad \text{(S6)}$$

$$r_{KC}^{(n)} = r_{KC}^{(n-1)} + 2\frac{D_{KC}}{r_{KC}^{(n-1)}}\Delta t + \sqrt{2D_{KC}\Delta t} W_{r,KC} \quad \text{(S7)}$$

As before, $W_{\theta,KC}$, $W_{\varphi,KC}$ and $W_{r,KC}$ are normally distributed random numbers, drawn independently and uncorrelated for each equation.

## 2. Implementation of boundary conditions for MT

In our model, both the angular motion and the growth of microtubules (MTs) are confined by the nuclear membrane. To impose this confinement we are using the following equations:

$$\theta_{MT} + \delta\theta_{MT} > \cos^{-1}\frac{L}{2R} \rightarrow \theta_{MT} = 2\cos^{-1}\frac{L}{2R} - (\theta_{MT} + \delta\theta_{MT}) \quad \text{(S8)}$$

$$\theta_{MT} > \cos^{-1}\frac{L+\delta L}{2R} \rightarrow \theta_{MT} = \cos^{-1}\left(\frac{L+\delta L}{2R}\right) \quad \text{(S9)}$$

where $\delta\Theta_{MT}$ and $\delta L$ denote the displacement that is computed when integrating the stochastic equations shown in figure 3B. The significance of these equations is the following: when a MT hits the boundary by pivoting (due to a change in one of the angular variables), it is reflected towards the center of the nucleus. If the tip of a MT grows until it touches the nuclear membrane (that is, the boundary is reached due to a change in $L$) it slides along it until it does not touch it anymore.

## 3. Discussion of the choice for the MT catastrophe rate

The theoretical results shown in the main text are obtained using a length-dependent MT catastrophe rate. Here, we discuss in more detail this choice and compare three possible models: (A) the length dependent catastrophe rate we used in the main text, (B) constant catastrophe rate and (C) catastrophes occurring when the MT tip is in the proximity of the boundary. For each of these models, we computed the MT length distribution and average kinetochore (KC) capture time and compared them to the experimental ones.

**A. Length dependent catastrophe rate (model of the main text).** In the model that we considered in the main text we assumed that MTs undergo catastrophe with a rate that increases linearly with their length:

$$k_{cat} = \alpha L \tag{S10}$$

In this case, an analytical approximation of the shape of the MT length distribution can be obtained for a simplified case. This is done starting from the equation:

$$\frac{\partial p(L)}{\partial t} = -\alpha L p(L) - v_g \frac{\partial p(L)}{\partial L} \tag{S11}$$

Equation S11 gives the variation in time of the probability density to have a growing MT of length $L$. The first term on the right hand side is the change in probability density due to the occurrence of catastrophes with a rate given by equation S10. The second one is the change in probability density due to MT growth. For simplicity, in writing equation S11 it was considered that the shrinking speed is much higher than the growth speed, so the amount of time that the MT spends in the

shrinking state can be neglected. The steady state probability density function to find a MT of length L is obtained by equating $\frac{\partial p(L)}{\partial t}$ to 0 and solving equation S11 for *p(L)*. We obtain:

$$p(L) = e^{-\frac{\alpha L}{2v_g}} \qquad \text{(S12)}$$

To test our model we used Monte Carlo simulations to predict the MT length distribution. The results of the simulations are plotted together with equation S12 and the experimental results inf S1B. We note that there can be MTs that are longer than the diameter of the cell and thus push the nuclear membrane towards the outside. However, this situation only occurs in approximately 4% of the simulation time.

The parameter $\alpha$ used in equation S10 was determined by linearly fitting the experimental catastrophe frequency, which was binned according to MT length. For this fit we have used equation S10 and not a two parameter equation of the type $k_{cat} = \alpha_0 + \alpha L$ in order to minimize the number of parameters in the model presented in the main text. Alternatively, the coefficient $\alpha$ could be computed by fitting the tail of the experimental length distribution with equation S12. We have also computed an apparent MT catastrophe probability as a function of length, over the entire simulation duration. It is shown in figure S1C and is in good agreement with the experiments.

**B. Constant catastrophe rate**. The simplest case would have been to consider a constant catastrophe rate. From the experimental data the catastrophe rate can be computed as:

$$k_{cat} = \frac{Total\ no\ of\ catastrophes}{\sum observed\ MT\ growth\ times} \qquad \text{(S13)}$$

We obtain the value $k_{cat}$=1.13± 0.62 min$^{-1}$. If one replaces the sum of observed growth times with that of the observed lifetimes the catastrophe rate becomes $k_{cat}$ =0.79 ± 0.32 min$^{-1}$. The MT length distribution in this case can be computed analytically and equals (4):

$$P(L) = \frac{1}{< L >} e^{-\frac{L}{<L>}} \qquad \text{(S14)}$$

where p is the probability of finding a MT with length $L$ and $<L>$ is the average MT length. The average MT length is connected to the catastrophe rate by (4):

$$< L > = \frac{v_s v_g}{v_s k_{cat} - v_g k_{res}} \tag{S15}$$

where $v_g$ is the MT growth velocity, $v_s$ is the MT shrink velocity, $k_{cat}$ is the catastrophe rate and $k_{res}$ is the rescue rate. In our case, because no rescue is observed experimentally, the catastrophe rate becomes:

$$k_{cat} = \frac{v_g}{< L >} \tag{S16}$$

Using the measured values of $v_g$ and $<L>$ we obtain a catastrophe rate of 1.59 $min^{-1}$.

We also computed the MT length distribution for a constant catastrophe rate using Monte Carlo simulations. The analytical, simulated and experimental length distributions are plotted together in figure S2A. It can be noticed that the analytical and simulated distributions have a longer and less steep tail than the experimental one. Also, in simulations we have the rather unrealistic situation that MTs reach a length larger than the diameter of the nucleus. In this situation, due to the boundary conditions that we use, they push through the nuclear membrane and pivoting is limited because of the constant interaction with the boundary (the MTs get “stuck” in the nucleus). Because this happens rather frequently (about 30 % of the simulation time) we decided to discard this model and investigate alternatives.

**C. Position dependent catastrophe rate (“boundary model”).** In the second alternative model catastrophes occur only when the MT tip is in an area of width $\sigma_{cat}$ in the vicinity of the nuclear membrane. The catastrophe rate, $k_{cat}$, is higher than in the other models. This condition writes as:

$$L > 2(R - \sigma_{cat}) \cos \theta_{MT} \tag{S17}$$

This model is based on previous observations (5) that MT growth can be stopped by the presence of an opposing force, in this case exerted by the nuclear membrane. The MTs that do not undergo catastrophe continue to grow and the boundary conditions are the same that we used in the main text. As in the previous models, it can happen that MTs grow longer than the size of the cell. In this case again, they continue to grow and stop pivoting, pushing through the cell membrane. Such a situation has also been observed experimentally for meiosis in fission yeast (6).This situation occurs in 6% of the simulated time. This description has the disadvantage that there are two parameters that have to be fitted: $\sigma_{cat}$ and $k_{cat}$. As before, we simulated the MT length distribution and compared it to the experimental one (figure S2B). We have also computed an apparent MT catastrophe probability as a function of length, over the entire simulation duration. It is shown in figure S2C and it similar to the experimental one increase to with the MT length, but not linearly.

Finally, we computed the average KC capture time in the case of 6 MT for all the three models described above. A comparison between all three models and the experimental data is shown in figure S2D.

All length distributions in figures S1 and S2 are computed including all MTs sizes. However, because not all MTs with a length smaller than 0.75 µm can be distinguished experimentally, the experimental probability density for those values could be underestimated.

### 4. Initial KC distribution

At the beginning of the simulation, the KCs are placed at a distance of $r_{KC} \approx 1.4$ µm from the spindle pole bodies, with the angular coordinates $\theta_{KC}$ and $\varphi_{KC}$ taking all possible values. This choice of coordinates means that the KCs are distributed on the surface region of a sphere with the center in the SPB and the radius equal to 1.4 µm that falls inside the nucleus. This choice was motivated by our experimental measurements of the initial distance between the KCs and the spindle pole bodies. It is moreover consistent with previous observations in the literature that at the onset of meiosis I in *S. pombe* KCs are far apart from the spindle pole bodies (7).

To understand how the initial distribution of KCs affects their capture times we performed a simulation in which their initial position was randomly picked according to a uniform distribution inside the nucleus. The fraction of free KCs as a function of time in this case is shown in figure S3, together with the experimental results and the prediction of the model of the main text. The capture process is slightly faster for the uniform distribution, due to the fact that now the KCs can be closer to the spindle pole bodies, so shorter MTs can capture them. That is, less time is lost due to MT growth.

**Table S1:** Strains used in this study

| **Name** | **Genotype** | **Source** |
|---|---|---|
| L972 | *WT, h-* | G. Rödel |
| L975 | *WT, h+* | G. Rödel |
| KI006 | *cdc25-22 -kanMX6-nmtP41-tdTomato-ndc80 leu1-32, h-* | Lab stock (1) |
| SV37 | *cdc25-22 sid4-GFP-kanMX -kanMX-nmtP3-GFP-atb2+, h-* | Lab stock (8) |
| SV54 | *GFP-atb2+ cut11-GFP sid4-GFP, h+* | Lab stock |
| MJ95 | *mCherry-atb2-hphMX leu1 ura4-D18, h-* | M. Sato |
| SI661 | *mCherry-sid4-natMX, h+* | S. Hauf |
| KI011 | *leu1-32 atb2-GFP sid4-GFP cut11-GFP ndc80::P41nmt-tdTomato-kanMX6, h+* | this study |
| GCAK1 | *mCherry-atb2-hphMX, ndc80-GFP-kanMX, h-* | this study |
| GC03 | *mCherry-atb2-hphMX ndc80-GFP-kanMX mCherry-sid4-natMX, h-* | this study |
| GC04 | *mCherry-atb2-hphMX ndc80-GFP-kanMX mCherry-sid4-natMX, h+* | this study |

**References**

1.Kalinina, I. *et al.* Pivoting of microtubules around the spindle pole accelerates kinetochore capture. *Nature cell biology* **15,** 82–87 (2012).

2.Gardiner, C. W. *Handbook of Stochastic Methods*. (Springer, 1985).

3.Chernykh, N. V. & Pakshin, P. V. Numerical Solution Algorithms for Stochastic Differential Systems with Switching Diffusion. **74,** 2037–2063 (2013).

4.Physical aspects of the growth and regulation of microtubule structures. *Phys Rev Lett* (1993).

5.Tischer, C., Brunner, D. & Dogterom, M. Force- and kinesin-8-dependent effects in the spatial regulation of fission yeast microtubule dynamics. *Mol Syst Biol* **5,** 1–10 (2009).

6.Kakui, Y., Sato, M., Okada, N., Toda, T. & Yamamoto, M. Microtubules and Alp7-Alp14 (TACC-TOG) reposition chromosomes before meiotic segregation. *Nature cell biology* **15,** 786–796 (2013).

7.Chikashige, Y. *et al.* Telomere-led premeiotic chromosome movement in fission yeast. *Science* **264,** 270–273 (1994).

8.Vogel, S. K., Raabe, I., Dereli, A., Maghelli, N. & Tolic-Norrelykke, I. Interphase microtubules determine the initial alignment of the mitotic spindle. *Current Biology* **17,** 438–444 (2007).

**Figure S1. A**. Average KC capture time as a function of the number of MTs computed using the model from the main text. The capture time is averaged over the duration that a KC pair spends diffusing in the nucleoplasm prior to being captured and over 10000 simulation runs. The average includes the last two pairs to be captured and the moment of capture of the first KC pair is taken as time zero. The thick horizontal line corresponds to the experimental value and the green shaded area shows the standard error of the mean. **B.** Experimental MT length distribution (green bars) compared to the simulated length distribution for the model used in the main text (MT catastrophe rate increases linearly with the MT length according to $k_{cat} = 1.15L$) (black bars). The black line shows the fit with the curve given by equation S12 with the parameters $\alpha$=1.15 min$^{-1}$ and $v_g$ = 2.4 µm/min. **C**. Experimental catastrophe rate as a function of MT length (green) compared to the length dependence computed from simulations made using the model in the main text (black). The error bars show the standard error of the mean.

**Figure S2. A.** Experimental MT length distribution (green bars) compared to the simulated length distribution for the case where the MT catastrophe rate is constant and equals $k_{cat}$ = 0.8 min$^{-1}$ (gray bars). The gray line shows the fit with the exponential curve given by equation S14 and $k_{cat}$=0.8 min$^{-1}$. **B**. Experimental MT length distribution (green bars) compared to the simulated length distribution for the case where catastrophes occur when the tip of the MT is in the vicinity of the nuclear boundary (gray bars). The parameters used in the simulations where $k_{cat}$=3.5 min$^{-1}$ and $\sigma_{cat}$=0.1 µm. **C.** Experimental catastrophe rate as a function of MT length (green) compared to the apparent length dependence computed from the simulation model in which catastrophes occur when the tip of the MT is in the vicinity of the nuclear boundary (gray). The parameters used in the simulations where $k_{cat}$=3.5 min$^{-1}$ and $\sigma_{cat}$=0.1 µm. The error bars show the standard error of the mean. **D**. Average KC capture times for the three models for MT catastrophe rate: $k_{cat} = 1.15L$ (model used in the main text, black), constant catastrophe rate (gray) and catastrophe occurring when the MT tip is in the proximity of the nuclear boundary (gray). For the constant catastrophe

rate the average capture time is shown for the two values discussed in the Supplementary note: $k_{cat}$=0.8 min$^{-1}$ and $k_{cat}$=1.6 min$^{-1}$.

**Figure S3.** Fraction of free KCs as a function of time for a uniform initial distribution of the KCs compared to the experimental results and simulations performed with the initial distribution of KCs used in the main text. Both simulations were done using 6MT and averaged over 10000 runs.

**Movie S1:** Paired movement of the KCs. Live cell microscopy of an *S. pombe* meiotic zygote, expressing tubulin labeled with mCherry (green), and the KC protein Ndc80 labeled with GFP (green); strains used KI0011and SV37 (Table S1). Time difference between frames is 3.45 s. Scale bar represents 1 µm. The movie corresponds to figure 1B & E.

**Movie S2:** MTs are highly dynamic. Live cell microscopy of an *S. pombe* meiotic zygote, expressing tubulin labeled with GFP (green), and the KC protein Ndc80 labeled with tdTomato (magenta); strains used GC003 and GC004 (Table S1). Time difference between frames is 3.5 s. Scale bar represents 1 µm. The movie corresponds to figure 2A.

**Movie S3:** MTs perform pivot around SPB. Live cell microscopy of an *S. pombe* meiotic zygote, expressing tubulin labeled with GFP (green), and the KC protein Ndc80 labeled with tdTomato (magenta); strains used GC003 and GC004 (Table S1). Time difference between frames is 4.3 s. Scale bar represents 1 µm. The movie corresponds to figure 2E.

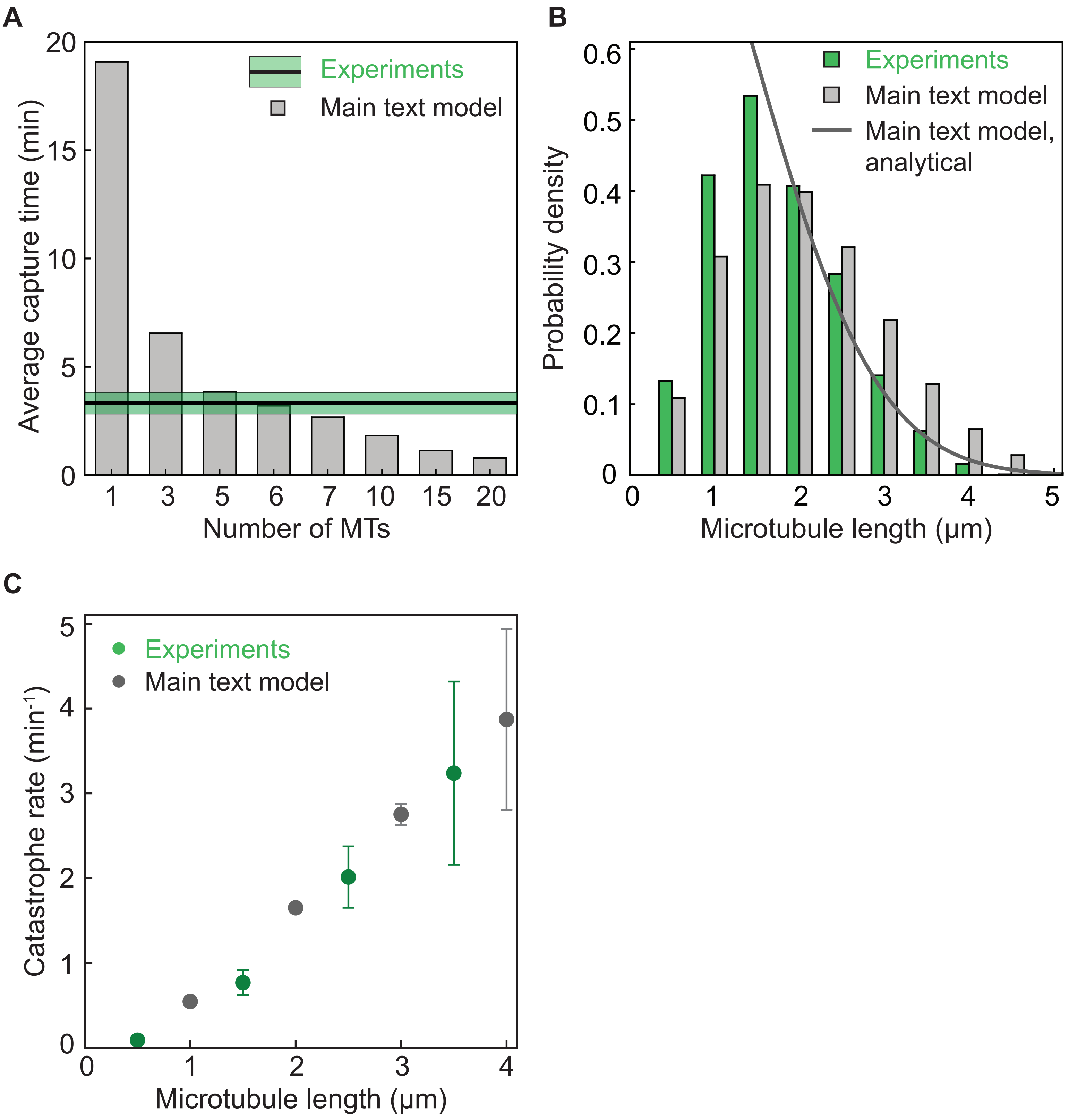

A
Experiments
Main text model
Average capture time (min)
Number of MTs
B
Experiments
Main text model
Main text model, analytical
Probability density
Microtubule length (μm)
C
Experiments
Main text model
Catastrophe rate (min$^{-1}$)
Microtubule length (μm)

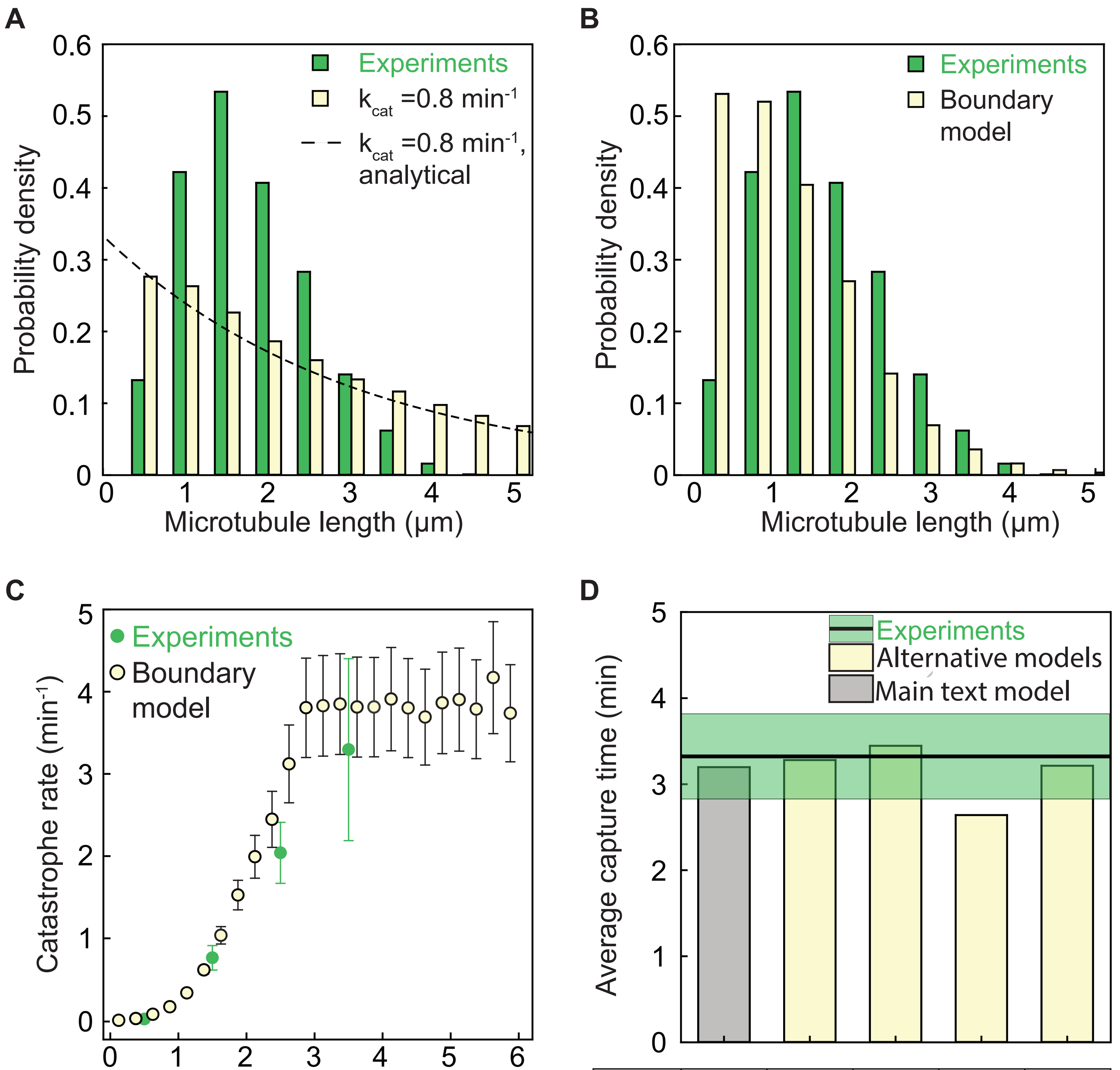


| | | | | | |
|---|---|---|---|---|---|
| $k_{cat}$ | 1.15L | 0.8 min$^{-1}$ | 1.6 min$^{-1}$ | 3.5 min$^{-1}$ | 3.5 min$^{-1}$ |
| n (MT) | 6 | 6 | 6 | 5 | 6 |
| $\sigma_{cat}$ | – | – | – | 0.1µm | 0.1µm |

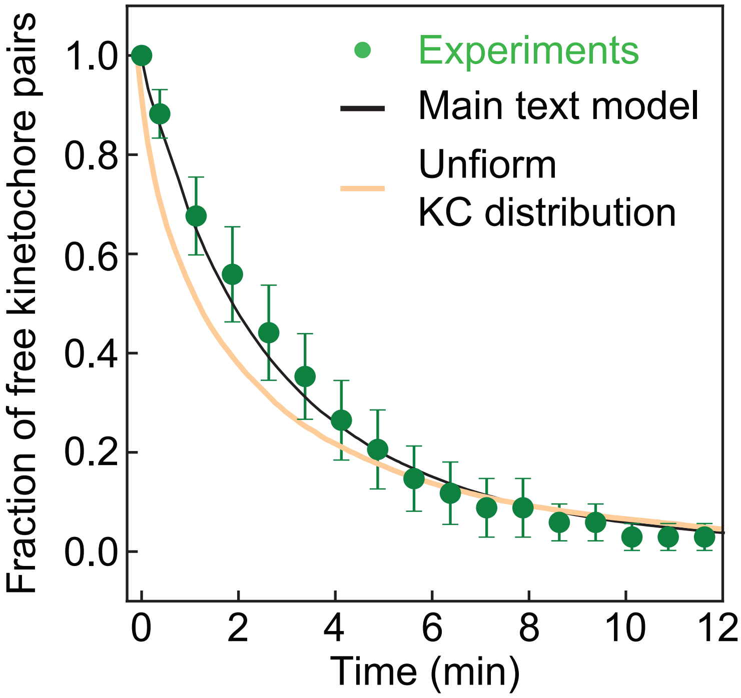
Experiments
Main text model
Unfiorm
KC distribution
Fraction of free kinetochore pairs
1.0
0.8
0.6
0.4
0.2
0.0
0
2
4
6
8
10
12
Time (min)
